# Immune-Boosting Effect of the COVID-19 Vaccine: Real-World Bidirectional Cohort Study

**DOI:** 10.2196/47272

**Published:** 2023-10-11

**Authors:** Ming Liu, Tianshuo Zhao, Qiuyue Mu, Ruizhi Zhang, Chunting Liu, Fei Xu, Luxiang Liang, Linglu Zhao, Suye Zhao, Xianming Cai, Mingting Wang, Ninghua Huang, Tian Feng, Shiguang Lei, Guanghong Yang, Fuqiang Cui

**Affiliations:** 1 Guizhou Center for Disease Control and Prevention Guiyang China; 2 Department of Laboratorial Science and Technology School of Public Health Peking University Beijing China; 3 Vaccine Research Center School of Public Health Peking University Beijing China; 4 Center for Infectious Diseases and Policy Research & Global Health and Infectious Diseases Group Peking University Beijing China; 5 Key Laboratory of Epidemiology of Major Diseases Peking University Ministry of Education Beijing China

**Keywords:** bidirectional cohort study, booster administration, COVID-19 vaccine, real-world study, SARS-CoV-2, vaccine efficacy, COVID-19

## Abstract

**Background:**

As the SARS-CoV-2 attenuates and antibodies from the COVID-19 vaccine decline, long-term attention should be paid to the durability of primary booster administration and the preventive effect of the second or multiple booster doses of the COVID-19 vaccine.

**Objective:**

This study aimed to explore the durability of primary booster administration and the preventive effect of second or multiple booster doses of the COVID-19 vaccine.

**Methods:**

We established a bidirectional cohort in Guizhou Province, China. Eligible participants who had received the primary booster dose were enrolled for blood sample collection and administration of the second booster dose. A retrospective cohort for the time of administration was constructed to evaluate antibody attenuation 6-12 months after the primary booster dose, while a prospective cohort on the vaccine effect of the second booster dose was constructed for 4 months after the second administration.

**Results:**

Between September 21, 2022, and January 30, 2023, a total of 327 participants were included in the final statistical analysis plan. The retrospective cohort revealed that approximately 6-12 months after receiving the primary booster, immunoglobulin G (IgG) slowly declined with time, while immunoglobulin A (IgA) remained almost constant. The prospective cohort showed that 28 days after receiving the second booster, the antibody levels were significantly improved. Higher levels of IgG and IgA were associated with better protection against COVID-19 infection for vaccine recipients. Regarding the protection of antibody levels against post–COVID-19 symptoms, the increase of the IgG had a protective effect on brain fog and sleep quality, while IgA had a protective effect on shortness of breath, brain fog, impaired coordination, and physical pain.

**Conclusions:**

The IgG and IgA produced by the second booster dose of COVID-19 vaccines can protect against SARS-CoV-2 infection and may alleviate some post–COVID-19 symptoms. Further data and studies on secondary booster administration are required to confirm these conclusions.

## Introduction

Since the end of 2019, COVID-19 has been the cause of a global pandemic, placing a heavy burden on the global public health system [1]. With the widespread and continuous evolution of SARS-CoV-2, many variants of concern (VOCs), such as Alpha (B.1.1.7), Beta (B.1.351), Gamma (P.1), Delta (B.1.617.2), and Omicron (B.1.1.529), have emerged globally and have led to several infection waves [2-5]. At present, the Omicron variant, which has a higher transmissibility and immune escape ability, is the dominant variant in the world [6]. Previous studies have shown that the Omicron variant not only has resistance to serum antibodies of convalescent patients but also has certain resistance to the serum of individuals who have been fully vaccinated against COVID-19 [7-12]. Therefore, Omicron poses a serious threat to the control of the COVID-19 pandemic and disease treatment.

China has administered 3.491 billion doses of the COVID-19 vaccine. The coverage rate of the first dose and second dose for the entire population reached 92.9% and 90.6%, respectively [13], and more than 771 million booster injections have been administered [14]. In November 2022, China adjusted and optimized the prevention and control measures for COVID-19. The local epidemic quickly climaxed, which led to a depletion of medical resources. Febrifuge, antitussive, and other COVID-19–related drugs could not meet the exponential increase in the number of patients in the short term, and the more serious concern was overwhelming the availability of hospital beds [15,16]. Therefore, long-term attention should be paid to the preventive effect and clinical value of the second or multiple booster doses of the COVID-19 vaccine.

In China, the most commonly used vaccines for both primary and booster immunization against COVID-19 are inactivated vaccines produced by the China National Biotec Group and Sinovac Biotech. Inactivated vaccines are prepared by cultivating SARS-CoV-2 in vitro to render the virus noninfectious while preserving its antigenicity. Although homologous boosting is generally considered a standard practice, heterologous regimens have been proposed as a COVID-19 vaccine strategy to elicit stronger and broader, or longer-lasting, immunity [17,18]. A recombinant COVID-19 vaccine using adenovirus type 5 as a vector for inhalation was developed by CanSino Biologics (inhalant Ad5-nCoV). Ad5-nCoV inhalation involves the recombination of the spike glycoprotein gene of SARS-CoV-2 into the replication-deficient human type 5 adenovirus gene, which induces an immune response in the body. This inhalant is easy to administer and can stimulate mucosal immunity. In a previous phase 1 trial, Ad5-nCoV inhalation was found to be well tolerated. Further, compared with intramuscular vaccination, aerosol vaccination could trigger a higher ratio of neutralizing antibodies to total antibodies [19].

Few real-world studies have demonstrated the effect of a fourth dose of heterologous booster on Omicron, especially using inhalation vaccines. Here, we aimed to reveal the immunogenicity and persistence of the primary booster dose and the real-world immune effect of the secondary inhalation booster to assess immunogenicity and persistence and prevent sequelae of booster administration in the real world.

## Methods

### Study Design

#### Overview

A bidirectional cohort was established to investigate the efficacy of booster administration between September 21, 2022, and January 30, 2023, in Guiyang City, Guizhou Province, China. The cohort was retrospectively tracked to determine the effect and durability of primary booster administration and prospectively followed up to assess the immunogenicity and real-world protective effect of secondary booster heterologous immunization, with the time of receiving the second booster as the node.

#### Retrospective Study

Blood samples were collected from individuals for antibody testing at the time of enrollment. A questionnaire covering basic characteristics and immunization programs was required to evaluate the durability and effectiveness of the third booster dose.

#### Secondary Booster Administration

All enrolled individuals received the inhalant, Ad5-nCoV, as part of a secondary booster immunization program (the fourth dose).

#### Prospective Study

About 4 weeks (21-35 days) after receiving the second booster dose, blood samples were collected for antibody testing. After about 16 weeks (84-140 days), information on the infection and sequelae of COVID-19 was collected from participants through follow-up phone calls.

### Participants

Participants were recruited by the Guizhou Center for Disease Control and Prevention. These individuals were aged 18 years or older and had received 3 doses of the COVID-19 vaccine before 6 months or above. The main exclusion criteria were individuals with a history of clinically or laboratory-confirmed COVID-19 or SARS-CoV-2 infection within the first 6 months of enrollment, a history of vaccination (any administration, including COVID-19 baseline or booster dose) within the first 6 months of enrollment, or an allergy to any component of the vaccine.

### Procedures

Individuals from Guanshanhu District, Qingzhen City, and Baiyun District of Guiyang City were recruited for this study. All individuals completed the basic and primary booster immunization procedures with the inactivated vaccine from the China National Biotec Group or Sinovac Biotech.

Eligible participants received 1 dose of inhalant Ad5-nCoV (0.1 mL per dose) through a specific atomization device. Venous blood samples (5 mL) were collected before inhalation and 28 days after inhalation to detect immunoglobulin G (IgG) and immunoglobulin A (IgA) antibodies against SARS-CoV-2 in serum. Antibody detection was performed by the receptor-binding domain antibody test kit produced by Vazyme Biotech Co Ltd. The kit detects receptor-binding domains IgA and IgG antibodies against SARS-CoV-2 that are produced during incubation through an indirect enzyme-linked immunosorbent assay (ELISA). After processing and color development, the absorbance of the sample was measured at a wavelength of 450 nm. The absorbance of the sample was positively correlated with the antibody titers.

### Survey Tool

Telephone follow-up was conducted with the participants to assess their status and the timing of contracting SARS-CoV-2 after inhalation and evaluate the persistent symptoms of post–COVID-19 using a scale. The scale comprised 49 items and was used to assess the severity of the post–COVID-19 impact using 8 indicators: fatigue, shortness of breath, brain fog, impaired coordination, physical pain, impaired sleep quality, depression, and impaired quality of life. These indicators were selected based on the common symptoms of post–COVID-19 condition (PCC), that is, a set of signs and symptoms that emerge during or after an infection consistent with COVID-19 and are not explained by an alternative diagnosis [20].

Each item was rated as “never occurred,” “slightly affected,” “moderately affected,” and “severely affected,” with scores of 0, 1, 2, and 3, respectively. The Cronbach α values of the 8 indicators ranged from .79 to .94, indicating acceptable reliability [21]. The detailed questionnaire and Cronbach α values are provided in Multimedia Appendix 1.

### Statistical Analyses

The baseline characteristics are presented as means (SDs) for continuous variables and percentages for categorical variables. Missing values were treated and reported in all analyses.

The attenuation curves of the antibody and time were fitted through locally weighted scatterplot smoothing (LOWESS), a nonparametric method used in the analysis of local regression. The sample was divided into short intervals, and weighted polynomial fitting to the sample in each interval was conducted. Linear regressions were constructed to fit the curve stratified according to the preceding immune program.

Comparisons of geometric mean titers (GMTs) and geometric mean increases (GMIs) between the groups were performed using logarithmic conversion values. The differences in antibodies before and after administration were compared using a paired Student 2-tailed *t* test, and linear regression was used to compare GMIs among different groups. For positive seroconversion, the antibodies after administration should increase by 4-fold or more, according to the literature [19,22]. A logistic regression was used to compare the seroconversion rate. On the basis of the marginal forecast rates of each category estimated by the regression, we used the weighted average of the standard population to calculate the direct standardized seroconversion rate and GMI. All test criteria to confirm the hypothesis were bilateral, with a significance level of .05. The adjusted α was reduced to .017 when pairwise comparisons were made between the 3 groups.

Kaplan-Meier analysis was used to plot the uninfected curves and cumulative hazard curves of different antibody levels, and the log-rank test was used to compare the difference in infection time among individuals with different levels. A multivariate Cox proportional hazard regression model was used to adjust for the effects of confounding factors on the results. Finally, a linear regression model was constructed to analyze the correlation between different sequelae scores and antibodies.

All statistical analyses were performed using R (version 4.2.0; R Development Core Team) and Stata (version 17.0; Stata Corporation).

### Ethics Approval

The protocol was approved by the institutional review board of the Guizhou Center for Disease Control and Prevention (approval number Q2023-03) and was performed in accordance with the Declaration of Helsinki and the Good Clinical Practice guidelines. All participants provided written informed consent before enrollment.

## Results

### Basic Characteristics of the Participants

A total of 327 participants who completed the vaccination and blood sampling were enrolled in the final statistical analysis. The specific participant entry and exit processes are outlined in Multimedia Appendix 1. A total of 234 female and 93 male participants were enrolled, with a mean age of 39.4 (SD 9.5) years. All participants received 3 doses of the COVID-19 vaccine before the survey, including 2 doses for basic immunization and 1 dose for booster immunization. Of the 327 participants, 166 received the BBIBP-CorV inactivated vaccine (SinoBio Pharmaceutical Ltd) and 161 received the CoronaVac inactivated vaccine produced by Sinovac Ltd. Among them, 233 participants worked in hospitals, 84 worked in nonhospital institutions, and the remaining 10 were reluctant to report their occupations and workplaces. Detailed information is provided in Table 1.

**Table 1:** The characteristics of participants (N=327).

Characteristics			Participants
Age (years), mean (SD)			39.4 (9.5)
**Age (years), n (%)**			
	<30	63 (19.3)	
	30-40	94 (28.7)	
	40-50	118 (36.1)	
	≥50	52 (15.9)	
**Sex, n (%)**			
	Male	93 (28.4)	
	Female	234 (71.6)	
**Ethnicity, n (%)**			
	Han	279 (85.3)	
	Minority	48 (14.5)	
**Workplace^a^, n (%)**			
	Hospital	233 (73.5)	
	Nonhospital	84 (26.5)	
**Primary booster vaccine**			
	BBIBP-CorV	166 (50.8)	
	CoronaVac	161 (49.2)	

^a^n=317; 10 subjects were not willing to report their occupation or workplace.

### Long-Term Durability of the Antibodies After Primary-Booster Administration (Third Dose)

**Figure 1:**
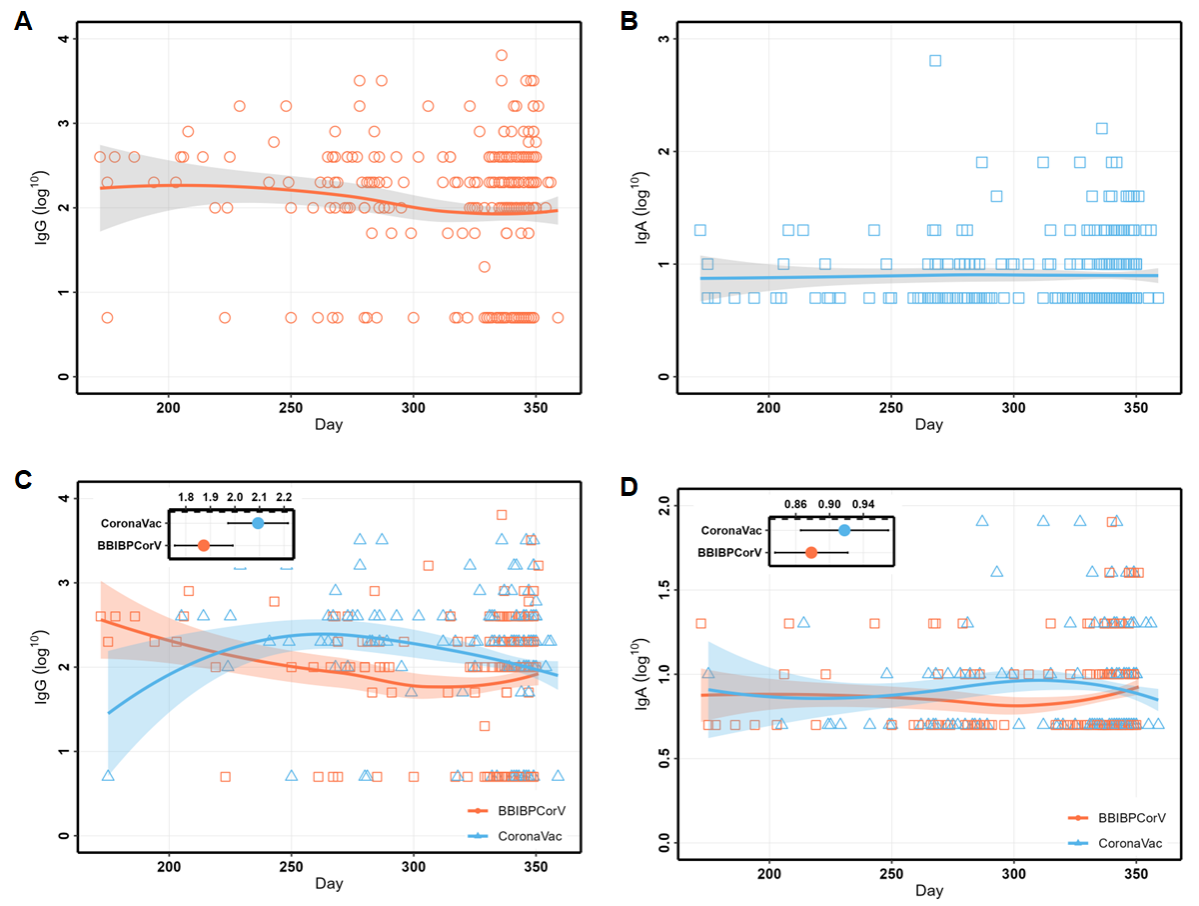
The locally weighted scatterplot smoothing (LOWESS) curves of IgG and IgA over time during 6-12 months after primary booster administration. (A) The curves of IgG over time. (B) The curves of IgA over time. (C) The changing curves of IgG stratified by a booster vaccine (third dose). (D) The changing curves of IgA stratified by a booster vaccine (third dose). IgA: immunoglobulin A; IgG: immunoglobulin G.

Before receiving the second booster (fourth dose), we measured the antibody levels of the participants. Approximately 6-12 months after receiving the booster, the GMT of the IgG antibody was 95.9 (95% CI 78.8-116.7), while that of the IgA antibody was 7.9 (95% CI 7.3-8.5). The time interval between the last booster immunization and antibody detection was used as the independent variable, while the antibody level was used as the dependent variable for LOWESS segmented curve fitting. The fitting results showed that the IgG antibody level slowly declined with time after the primary booster, while the IgA antibody level remained almost constant (GMT 7.9; Figure 1A and B).

We proceeded to stratify the results based on the different immunization programs and perform linear fitting. Figure 1C shows that the declining trend for the IgG antibody level of participants administered the CoronaVac vaccine and those administered the BBIBP-CorV vaccine was consistent after 6 months of immunization, with the IgG antibody titer of CoronaVac (GMT 124.2; 95% CI 93.8-164.3) vaccine recipients being slightly higher than that of the BBIBP-CorV vaccine recipients (GMT 74.7; 95% CI 56.9-97.9). Figure 1D shows that the levels and trends of the 2 are almost identical.

### Immunogenicity of the Second Booster (Fourth Dose)

At 28 days after receiving the inhalant vaccine as the second booster, the GMT of the IgG antibody was 5066.5 (95% CI 4418.1-5810.1), while that of the IgA antibody was 108.6 (95% CI 95.4-123.5). The GMI of IgG was 52.8 (95% CI 42.6-65.6), and the seroconversion rate reached 94.5% (95% CI 92-97). The GMI of IgA was 13.7 (95% CI 12-15.7), and the antibody seroconversion rate was 89.3% (95% CI 85.9-92.7).

**Figure 2:**
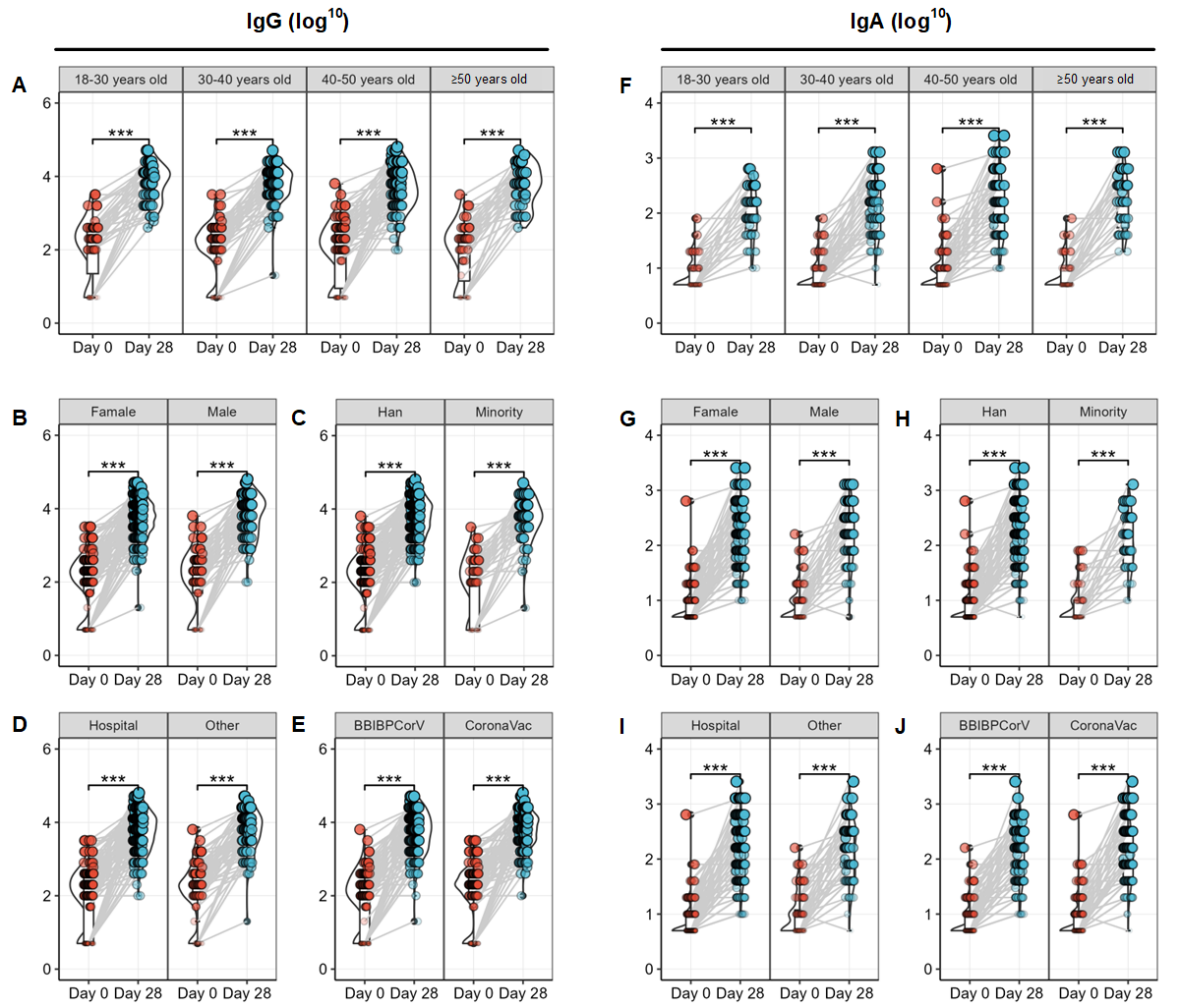
The differences of the pre- and postvaccination GMTs for the IgG and IgA antibodies of different groups. (A-E) GMTs for the IgG. (A) Group by age. (B) Group by sex. (C) Group by ethnicity. (D) Group by type of primary booster vaccine. (E) Group by workplace. (F-J) GMTs for the IgA. (F) Group by age. (G) Group by sex. (H) Group by ethnicity. (I) Group by type of primary booster vaccine. (J) Group by workplace. GMT: geometric mean titer; IgA: immunoglobulin A; IgG: immunoglobulin G.

The antibody levels of individuals with different sociodemographic characteristics and prevaccination programs showed significant improvement after vaccination. The line graphs of the pre and postvaccination GMTs for the IgG and IgA antibodies of different groups are shown in Figure 2.

We explored the crude and adjusted changes in antibody (GMI and seroconversion rate) among different groups based on age, sex, ethnicity, workplace, and type of primary booster vaccine, as detailed in Table 2. There was no difference in postvaccination GMI or seroconversion of IgG antibodies among the different demographic groups. However, for participants who received BBIBP-CorV (adjusted rate 97.4%; 95% CI 94.9-99.9) as their initial booster, the seroconversion rate of the IgG antibody was higher than that of those who received CoronaVac (adjusted rate 91.4%; 95% CI 87.2-95.7) after the secondary booster immunization.

**Table 2:** The distribution and difference of geometric mean increase and positive seroconversion rate of immunoglobulin G and immunoglobulin A antibodies among groups.

Variable				Geometric mean increase, mean (95% CI)							Seroconversion rate, proportion (95% CI)				
				Crude		Adjusted		*P* value		Crude			Adjusted		*P* value
**Immunoglobulin G**															
	Overall			52.8 (42.6-65.6)		N/A^a^		N/A		94.5 (92-97)			N/A		N/A
	**Age (years)**														
		<30	56.6 (33.9-94.7)		53.6 (32.2-89.1)		—^b^		93.7 (87.6-99.7)			92.3 (85-99.7)		—	
		30-40	49.1 (35.1-68.6)		52.2 (34.4-79.3)		.94		97.9 (94.9-100.8)			97.7 (94.5-100.9)		.16	
		40-50	48.4 (32.2-72.6)		49.9 (34.3-72.5)		.83		91.5 (86.5-96.6)			92.3 (87.6-96.9)		.99	
		≥50	67.7 (41-111.9)		69.8 (39.8-122.7)		.49		96.2 (90.9-101.5)			95.9 (90.5-101.4)		.45	
	**Sex**														
		Male	52 (34.6-78.1)		57.6 (37.4-88.8)		—		92.5 (87.1-97.9)			93.3 (88.3-98.2)		—	
		Female	53.2 (41.2-68.6)		52.6 (40.3-68.6)		.73		95.3 (92.6-98)			94.8 (91.8-97.8)		.59	
	**Ethnicity**														
		Han	52.8 (41.9-66.5)		53.5 (42.1-68)		—		94.3 (91.5-97)			94.2 (91.5-96.9)		—	
		Minority	52.9 (28.6-97.9)		56.8 (31.6-102.1)		.85		95.8 (90.1-101.6)			95 (88.3-101.7)		.84	
	**Workplace**														
		Hospital	61.3 (47-80.1)		61.5 (47.4-79.8)		—		94.4 (91.5-97.4)			94.4 (91.5-97.3)		—	
		Nonhospital	37.9 (26-55.1)		37.6 (24.2-58.4)		.06		94 (88.9-99.2)			94.1 (89-99.2)		.92	
	**Booster vaccine**														
		BBIBP-CorV	64.1 (48-85.6)		65.7 (48-89.9)		—		97.6 (95.2-99.9)			97.4 (94.9-99.9)		—	
		CoronaVac	43.3 (31.4-59.6)		44.3 (32.4-60.7)		.09		91.3 (86.9-95.7)			91.4 (87.2-95.7)		.03	
**Immunoglobulin A**															
	Overall			13.7 (12-15.7)		N/A		N/A		89.3 (85.9-92.7)			N/A		N/A
	**Age (years)**														
		<30	12.9 (9.8-16.8)		13.2 (9.7-18)		—		88.9 (81-96.7)			89.2 (81.5-96.9)		—	
		30-40	14.1 (11.2-17.7)		14.8 (11.5-19.1)		.58		92.6 (87.2-97.9)			93.7 (88.8-98.6)		.31	
		40-50	11.8 (9.3-14.9)		11.1 (8.9-14)		.38		83.9 (77.2-90.6)			82.5 (75.3-89.8)		.25	
		≥50	20.3 (14.6-28.2)		20.4 (14.5-28.7)		.07		96.2 (90.9-101.5)			95.9 (90.3-101.5)		.21	
	**Sex**														
		Male	16.5 (12.6-21.5)		16.9 (13-21.9)		—		89.2 (82.9-95.6)			89.7 (83.4-95.9)		—	
		Female	12.8 (11-14.8)		12.6 (10.8-14.9)		.07		89.3 (85.3-93.3)			89.1 (85-93.2)		.88	
	**Ethnicity**														
		Han	13.9 (12.1-16)		13.8 (11.9-15.9)		—		90.3 (86.8-93.8)			90.1 (86.6-93.6)		—	
		Minority	12.8 (8.6-19.2)		13.6 (9.5-19.3)		.94		83.3 (72.6-94)			83.9 (73.2-94.7)		.22	
	**Workplace**														
		Hospital	14 (12-16.4)		14.3 (12.2-16.7)		—		89.7 (85.8-93.6)			89.8 (86-93.6)		—	
		Nonhospital	13 (9.8-17.1)		12.3 (9.4-16.1)		.36		88.1 (81.1-95.1)			87.8 (80.6-94.9)		.62	
	**Booster vaccine**														
		BBIBP-CorV	13.3 (11-15.9)		13.1 (10.8-15.8)		—		88.6 (83.7-93.4)			87.6 (82.5-92.8)		—	
		CoronaVac	14.2 (11.8-17.2)		14.4 (11.9-17.5)		.48		90.1 (85.4-94.7)			90.8 (86.4-95.2)		.36	

^a^N/A: not applicable.

^b^—: not available.

### Real-World Protective Effect of Secondary Booster Administration Against Infection

After the second booster, we continued to track and follow up with the participants to derive the breakthrough infection rates. As of January 30, 2022, we followed up with 251 participants; the breakthrough infection rate was 60.2% (151/251).

**Figure 3:**
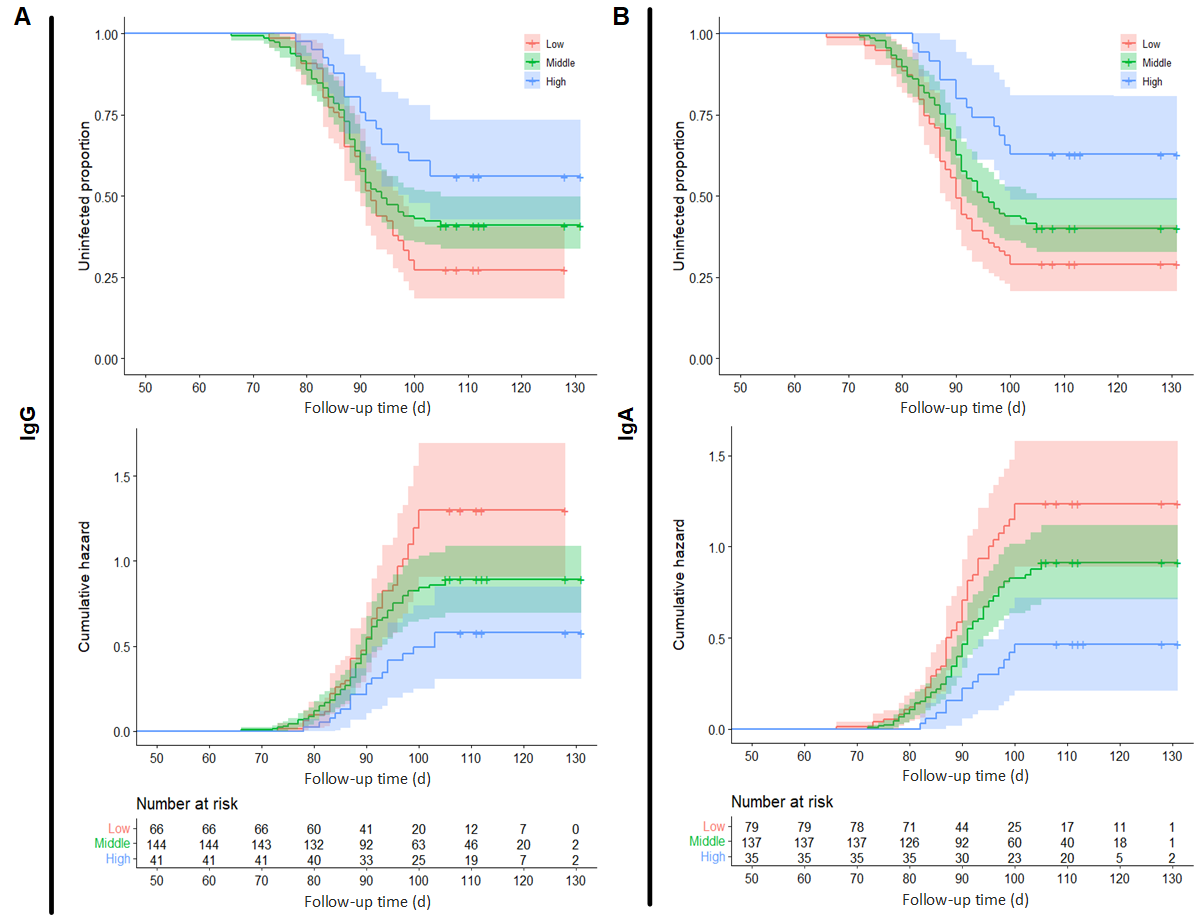
The Kaplan-Meier and cumulative hazard curve of COVID-19 infection grouped by antibody levels. (A) Curves grouped by IgG levels (low, middle, and high). (B) Curves grouped by IgA levels (low, middle, and high). IgA: immunoglobulin A; IgG: immunoglobulin G.

After adjusting for confounding factors, Cox regression analysis revealed a significant correlation between postimmunization IgG antibody levels and breakthrough COVID-19 infection (hazard ratio 0.60; 95% CI 0.45-0.79); the same result was obtained for IgA antibody levels (hazard ratio 0.55; 95% CI 0.39-0.78). This result suggests that both IgG and IgA antibodies after the second booster can provide a certain degree of protection against COVID-19 and prevent infection. We categorized postimmunization IgG and IgA antibodies into 3 levels, high, medium, and low, based on 25th percentile and 75th percentile. Survival and cumulative risk curves were plotted for each level, as shown in Figure 3. Higher levels of postimmunization IgG antibodies were associated with better protection against COVID-19 infection and a lower cumulative risk for vaccine recipients (Figure 3A). Similarly, higher levels of postimmunization IgA antibodies were associated with better protection against COVID-19 infection and a lower cumulative risk for vaccine recipients (Figure 3B).

Kaplan-Meier curves for the risk of COVID-19 infection for age and sex subgroups were plotted, as shown in Figure 1. Differences between age groups were found: participants between 30 and 40 years of age had a higher hazard risk than other age groups. The differences were insignificant after adjusting for other sociodemographic factors. Women appear to have a higher risk of infection than men, but this did not reach statistical significance (*P*=.051).

### Real-World Protective Effect of Secondary Booster Administration Against Post–COVID-19 Symptoms

Finally, a questionnaire was used to evaluate the post–COVID-19 symptoms of 151 participants who experienced breakthrough infections, including fatigue, shortness of breath, brain fog, impaired coordination, physical pain, impaired sleep quality, depression, and impaired quality of life. The most common symptoms were fatigue, impaired sleep quality, and impaired quality of life, with mean scores of 0.46, 0.24, and 0.20, respectively.

**Figure 4:**
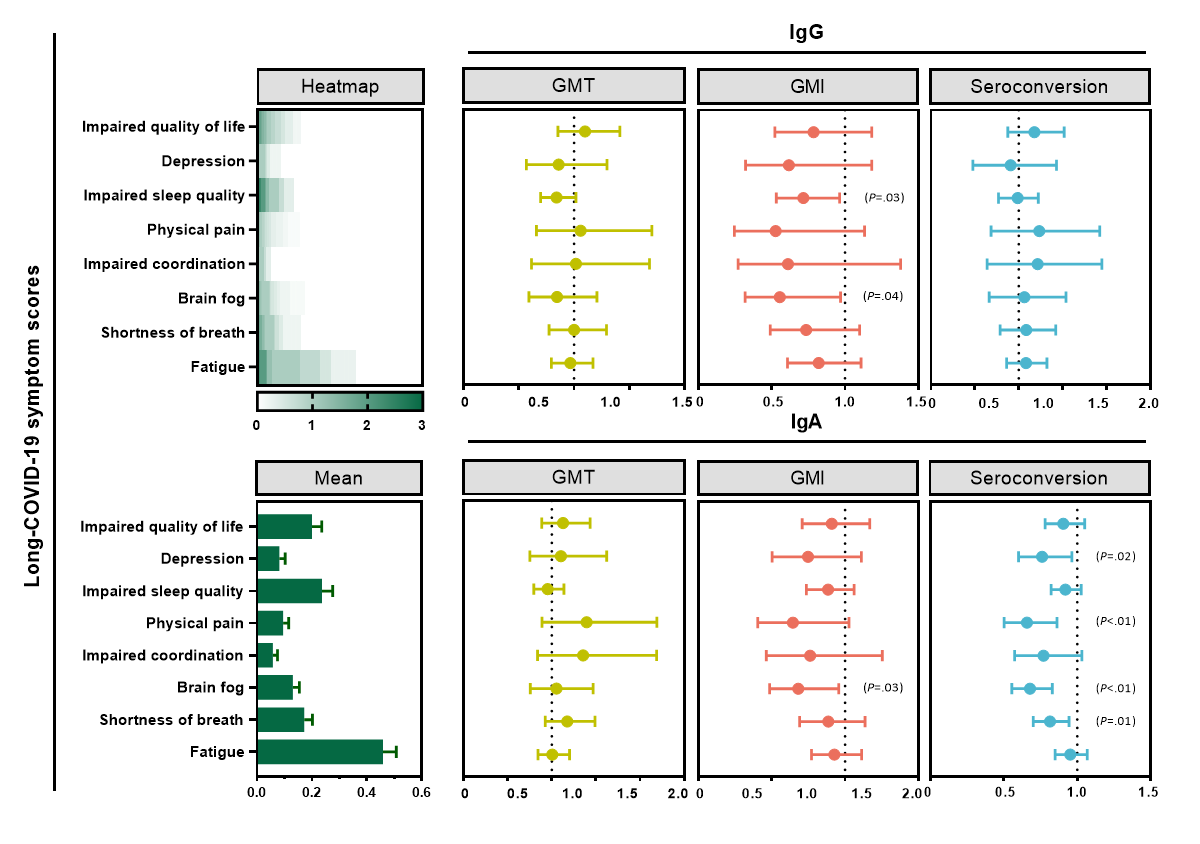
The adjusted regression models of antibodies on post–COVID-19 scores. The heatmap and bar chart on the left represent the discrete and central trends of the post–COVID-19 scores, and the scores of each subitem entered the model as a dependent variable. The 3 graphs on the top right are the results of entering different metrics of IgG (ie, GMT, GMI, and seroconversion rate) into the regression model as independent variables. The 3 graphs on the bottom right are the results of entering different metrics of IgA (ie, GMT, GMI, and seroconversion rate) into the regression model as independent variables. Each regression adjusted for basic sociodemographic characteristics. GMI: geometric mean increase; GMT: geometric mean titer; IgA: immunoglobulin A; IgG: immunoglobulin G.

We constructed a regression model with postimmunization antibody levels as the independent variable and questionnaire scores as the dependent variable. Based on the results, the GMI levels of postimmunization IgG antibodies had a protective effect on brain fog (odds ratio [OR] 0.56; 95% CI 0.32-0.97) and sleep quality (OR 0.71; 95% CI 0.53-0.96). The GMI levels of postimmunization IgA antibodies had a protective effect on brain fog (OR 0.68; 95% CI 0.49-0.96). Additionally, participants who were IgA antibody seropositive after infection had milder symptoms of shortness of breath (OR 0.81; 95% CI 0.7-0.95), brain fog (OR 0.68; 95% CI 0.55-0.83), impaired coordination (OR 0.66; 95% CI 0.50-0.86), and physical pain (OR 0.76; 95% CI 0.60-0.96; Figure 4).

The age-stratified protective effect of IgG and IgA after secondary enhancement on post–COVID-19 symptoms was reported. Unfortunately, no differences between age groups were found, and detailed results can be seen in Figure 2.

## Discussion

### Overview

Based on existing evidence, there is a significant decline in neutralizing antibody titers 4-5 months after completion of the routine vaccination program [23,24]. The immune protection induced by the vaccine declines continuously with time, highlighting the urgent need for booster vaccination to enhance protection. According to statistics from the National Health Commission, approximately 850 million people in China have received their booster vaccine as of February 2023 [16]. The administration of a third dose of the same vaccine has been demonstrated to significantly increase neutralizing antibody levels and effectively reduce the symptomatic infection rate of the SARS-CoV-2 variant [25,26].

The literature suggests a decline or even disappearance of antibody levels within a short period of 3-6 months [27-30]. In this study, we tracked the antibody level owing to the first booster for 6-12 months without the interference of natural infection to evaluate long-term immunogenicity. We found that the decline in antibody level was slow after 6 months and maintained at a relatively low level, with a GMT of approximately 96 for IgG and approximately 8 for IgA. In confirmatory research, vaccine effectiveness was estimated to decline from approximately 70% one week after the booster dose to approximately 40% at 15 weeks or more [31].

The titers of postadministration antibodies vary according to the vaccine type. In China, inactivated vaccines are the most commonly used vaccines for basic and booster immunizations owing to their safety and stability. Based on evidence from the Chinese Center for Disease Control and Prevention, the GMTs owing to BBIBP-CorV were 25 at 1 month and 4 at 12 months, while those owing to CoronaVac were 20.2 and 4.1, respectively [32]. In this study, ELISA revealed that the IgG antibody titers from CoronaVac were slightly higher than those from BBIBP-CorV at 6-12 months after administration but converged at 12 months.

Breakthrough infections have become more common with the decline in antibody levels and the development of new VOCs with strong immunologic escape, despite the remarkable effect of primary boosting [33]. A fourth dose of the COVID-19 vaccine can boost cellular and humoral immunity, and the peak responses were found to be similar to the peak responses after the third dose [34]. According to some clinical trials, the adenovirus vector booster dose based on an inactivated vaccine could lead to higher neutralization antibodies than homologous boostering [35,36]. In China, nearly 47 million residents have now completed the sequential booster immunization since the start of its dissemination in November 2022 [14].

Based on the available evidence, a prospective study was conducted to evaluate heterologous secondary booster administration. To our knowledge, this study is the first real-world evaluation of the effectiveness of the second booster dose in China. Herein, the inhalant Ad5-nCoV vaccine was administered as a second booster dose. Inhalant Ad5-nCoV is homologous to injectable Ad5-nCoV but achieves protection through mucosal immunization through inhalation. Mucosal immunity is a critical component of the human immune system, with more than 90% of infections occurring in the mucosa, which comprises numerous dendritic cells with strong T-cell activation capacity that can induce an immune response. ELISA to detect the serum antibodies revealed that the GMT of the IgG antibody was 4978.2 and that of the IgA antibody was 107.8 at 28 days after inhalation. The seroconversion rates for IgG and IgA were 93.8% and 86.9%, respectively. The antibody titers of the primary booster against the Omicron variant were attenuated relative to those of other virus strains, such as the wild type and other VOCs. Therefore, the use of heterogeneous vaccines for the second-booster procedures is a concern for the prevention of the Omicron variant [37].

China suffered a COVID-19 epidemic between December 2022 and January 2023 owing to changes in health policies and the impact of the Omicron variant, with a peak in cases on December 22, 2022 [13]. In this study, we followed up with participants for 4 months after receiving their fourth vaccine dose to assess their real-world COVID-19 infection status. As of January 30, 2023, 60.2% (151/251) of participants self-reported that they had been infected with COVID-19. Notably, IgG and IgA provided strong protection against COVID-19 infection, as demonstrated by the high antibody titers after the fourth dose.

In addition to infection prevention, the long-term effects of COVID-19 are also concerning. Studies from high-income countries suggest that vaccination may alleviate post–COVID-19 or PCC [38]. However, based on other evidence, COVID-19 vaccination is not associated with improvement in PCC [39,40]. We sought to assess some nonspecific post–COVID-19 symptoms. Based on our results, the GMI and seroconversion rates of IgG and IgA may alleviate some of the symptoms after secondary booster administration, including sleep quality, shortness of breath, brain fog, impaired coordination, and physical pain. The GMTs had no statistical relationship, which may be explained by the insufficient statistical efficacy induced by the small sample size. However, its clinical value is still worth exploring. The varying levels of IgG and IgA suggest the mechanisms of immune protection after infection, suggesting that further tracking and research are warranted.

Our study had some limitations. First, the SARS-CoV-2 infection of participants was self-reported, which may have led to recall and reporting biases. Further, asymptomatic patients may not have been identified. Second, the antibody detection method used was an ELISA quantitative assay rather than the neutralization test. However, the results of both tests are highly correlated according to the literature. ELISA can be used as a substitute for the gold standard to assess immunogenicity [22]. Third, there was no control group setting without a second booster dose for estimating the vaccine effect of the second booster dose. Their use of antibody levels was reasonable in accordance with previous trials, however [41]. Finally, only follow-up data collected within approximately 1 month after infection were reported in this study. These findings may offer suggestions for future populations with post–COVID-19. Further studies are needed, including appropriate control measures for unvaccinated individuals, to confirm the trajectory of persistent symptoms after COVID-19 vaccination.

### Conclusions

The IgG and IgA antibodies did not decrease significantly but remained at a relatively low level after administration of the second booster dose. The antibodies generated significant immunogenic protection against breakthrough infections and might partially alleviate post–COVID-19 symptoms. Further studies on secondary booster administration are needed to validate these correlations.

## Appendix

Multimedia Appendix 1 Post-COVID symptom scale, Kaplan-Meier curves of COVID-19 infection, and age-stratified correlation between post-administrated antibody and post-COVID symptoms.

## Abbreviations

ELISA: enzyme-linked immunosorbent assay
GMI: geometric mean increase
GMT: geometric mean titer
IgA: immunoglobulin A
IgG: immunoglobulin G
LOWESS: locally weighted scatterplot smoothing
OR: odds ratio
PCC: post–COVID-19 condition
VOC: variant of concern

## Data Availability

All data sets generated during and/or analyzed during this study are available from the corresponding author on reasonable request.
