# Supplementary material for: Immune-Boosting Effect of the COVID-19 Vaccine: Real-World Bidirectional Cohort Study
Source: JMIR Public Health Surveill. 2023 Oct 11;9:e47272. doi: 10.2196/47272 (PMC10569382; doi:10.2196/47272)
Supplement: Multimedia Appendix 1 [file publichealth_v9i1e47272_app1.docx]

**Supplemental Materials**

**Supplemental Table 1. Post-COVID symptom scale**

| **Symptom** | **Item** | **Score** | **Cronbach'α** |
| --- | --- | --- | --- |
| Fatigue | |  | 0.907 |
|  | Do you feel physically and mentally exhausted, or feel unenergetic despite resting | 0-3 |  |
|  | Are you interested in somethings but feel mentally unwell or lack energy to do it | 0-3 |  |
|  | Do you feel less energetic or are you unable to support your physical strength after resting or sleeping | 0-3 |  |
|  | Do you feel more physically fatigued and mentally exhausted after simple activities or mental work | 0-3 |  |
| Shortness of breath | |  | 0.914 |
|  | Do you experience short of breath or have difficulty breathing when sitting in a normal position | 0-3 |  |
|  | Do you experience short of breath or have difficulty breathing when walking upstairs or climbing stairs | 0-3 |  |
|  | Do you experience short of breath when lying flat | 0-3 |  |
|  | Do you experience chest compression or discomfort breathing when breathing | 0-3 |  |
|  | Do you experience short of breath when breathing normally through nose | 0-3 |  |
| Brain Fog | |  | 0.930 |
|  | Do you have difficulty remembering things or do you forget things you should do | 0-3 |  |
|  | Do you often forget things or can't remember common things | 0-3 |  |
|  | Does you feel foggy, dull or jet-lagged, or experience mind blanking | 0-3 |  |
|  | Do you feel confused about what is happening around you | 0-3 |  |
|  | Do you find it difficult to concentrate on work or thinking | 0-3 |  |
|  | Do you find it difficult to plan your work or follow your plan | 0-3 |  |
|  | Do you find it difficult to find the right words to express yourself verbally or in writing | 0-3 |  |
|  | Do you find it difficult to understand what others are saying or the meaning behind it | 0-3 |  |
|  | Do you utter unclear or incoherent speech | 0-3 |  |
|  | Do you experience difficulty reading | 0-3 |  |
| Impaired coordination | |  | 0.785 |
|  | Do you have uncontrollable tremors or involuntary movements in parts of your body | 0-3 |  |
|  | Do you have trouble balancing your body | 0-3 |  |
|  | Do you have trouble coordinating your body or limbs | 0-3 |  |
| Physical Pain | |  | 0.920 |
|  | Do you experience any chest pain | 0-3 |  |
|  | Do you experience any pain when breathing | 0-3 |  |
|  | Do you experience any dull or stabbing pain | 0-3 |  |
|  | Do you experience any pulling or burning pain | 0-3 |  |
|  | Do you experience any muscle pain | 0-3 |  |
|  | Do you experience any muscle weakness | 0-3 |  |
|  | Do you experience any muscle stiffness | 0-3 |  |
|  | Do you experience any joint pain | 0-3 |  |
|  | Do you experience any joint swelling | 0-3 |  |
|  | Do you experience any joint stiffness | 0-3 |  |
|  | Do you experience any muscle spasms | 0-3 |  |
|  | Do you experience any muscle cramps | 0-3 |  |
|  | Do you feel any tingling (like pins and needles) or numbness in your limbs | 0-3 |  |
| Impaired sleep quality | |  | 0.839 |
|  | Do you have difficulty falling asleep | 0-3 |  |
|  | Is your sleep time shorter than before (before COVID-19) | 0-3 |  |
|  | Is your sleep interrupted or disturbed | 0-3 |  |
| Depression | |  | 0.886 |
|  | Are you uninterested or bored with everything around you | 0-3 |  |
|  | Do you experience anxiety | 0-3 |  |
|  | Do you feel sad or miserable | 0-3 |  |
|  | Do you experience emotional fluctuations | 0-3 |  |
|  | Do you feel lonely or helpless | 0-3 |  |
| Impaired quality of life | |  | 0.944 |
|  | Does your symptom affect your ability to go shopping or travel | 0-3 |  |
|  | Does your symptom affect your ability to do household chores | 0-3 |  |
|  | Does your symptom affect your ability to engage in light activity | 0-3 |  |
|  | Does your symptom affect your ability to take care of yourself, such as bathing or getting dressed | 0-3 |  |
|  | Does your symptom affect your relationship with family members or friends | 0-3 |  |
|  | Does your symptom affect your social activities and interactions with others | 0-3 |  |


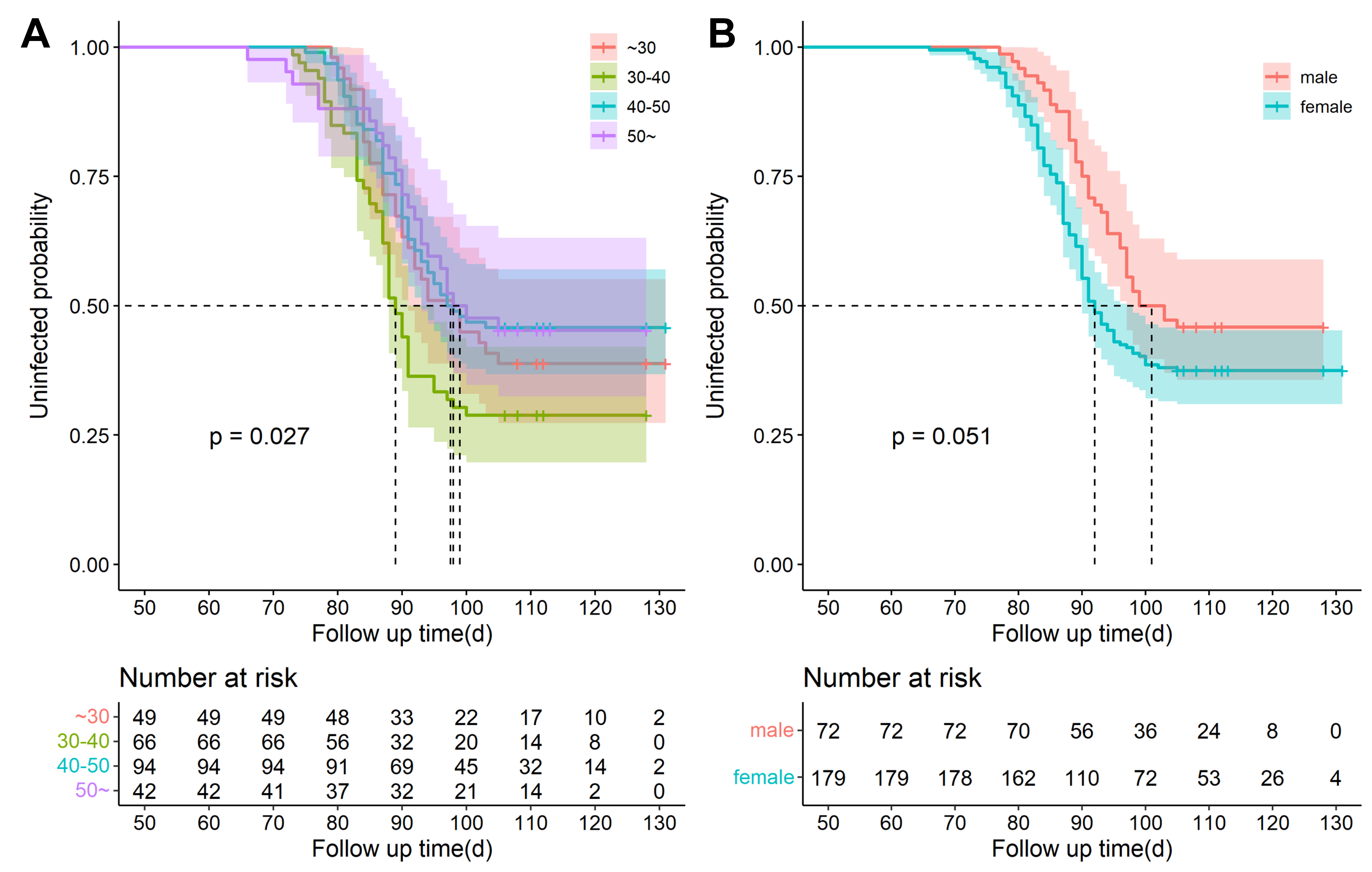


Supplemental Figure 1. The Kaplan-Meier curves of COVID-19 infection in different age and sex categories over time after secondary booster. (A) Curves grouped by age; (B) Curves grouped by sex.


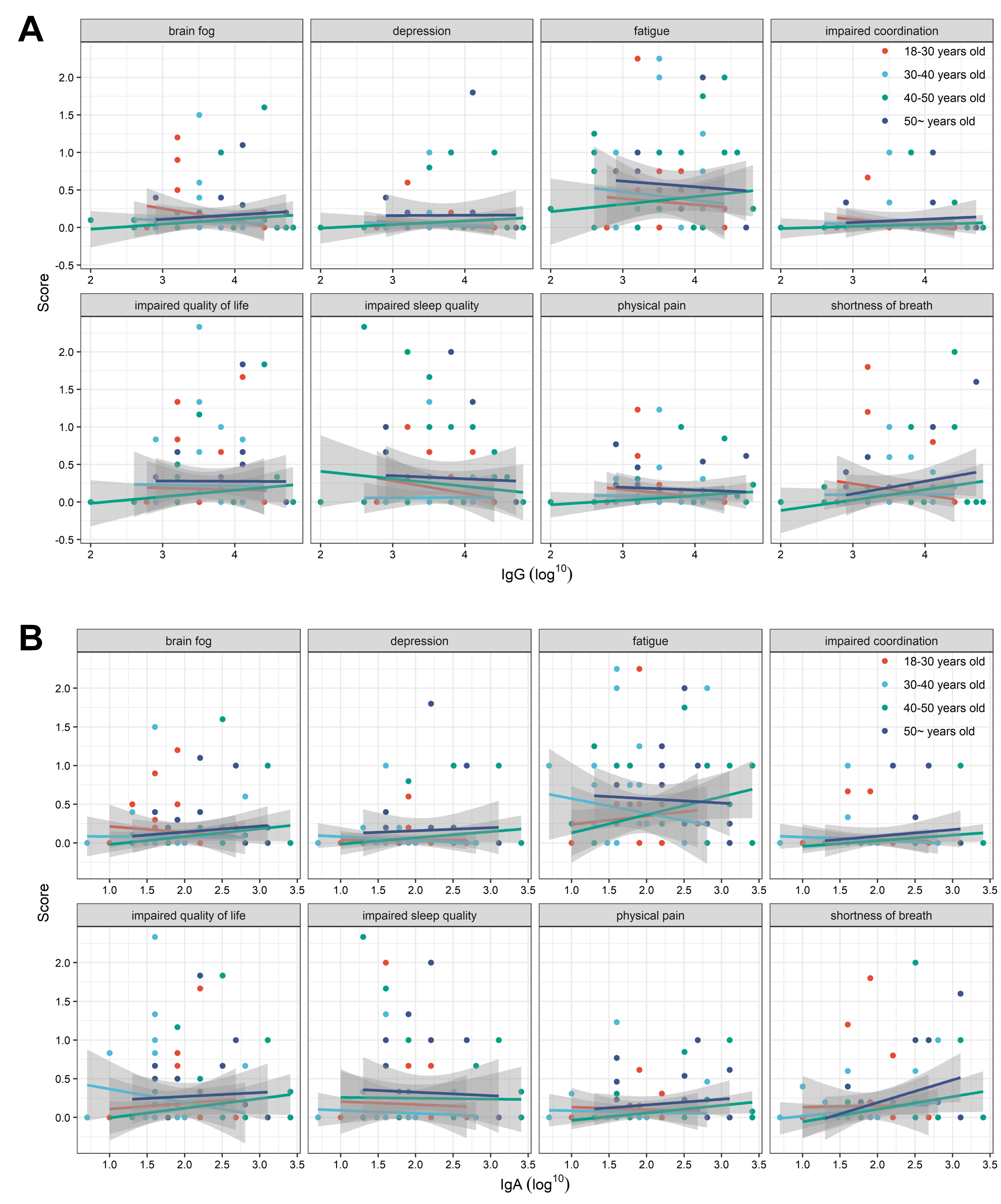


Supplemental Figure 2. The age-stratified correlation between post-administrated antibody and post-COVID symptoms. (A) Fitted curve between post-administrated IgG and post-COVID symptom; (B) Fitted curve between post-administrated IgG and post-COVID symptom.
